# Seroprevalence and risk factors of bluetongue virus in domestic cattle, sheep, goats and camels in Africa: a systematic review and meta-analysis

**DOI:** 10.1080/01652176.2024.2396118

**Published:** 2024-08-30

**Authors:** Bachir Medrouh, Amine Abdelli, Salem Belkessa, Yacine Ouinten, Malika Brahimi, Ahcène Hakem, Tahar Kernif, Steven M. Singer, Hocine Ziam, Anastasios D. Tsaousis, Pikka Jokelainen, Giovanni Savini, Edoardo Pasolli

**Affiliations:** a Research Centre for Agropastoralism, Djelfa, Algeria; b Department of Agricultural Sciences, University of Bouira, Bouira, Algeria; c Laboratory of Exploration and Valorization of Steppic Ecosystems, Department of Biology, Faculty of Nature and Life Sciences, Ziane Achour University of Djelfa, Djelfa, Algeria; d Laboratory of Parasitic Eco-epidemiology and Population Genetics, Pasteur Institute of Algeria, Dely-Brahim, Algeria; e Department of Biology, Georgetown University, Washington, DC, USA; f Laboratory of Biotechnology, Environment and Health, University of Blida 1, Blida, Algeria; g Laboratory of Molecular & Evolutionary Parasitology, RAPID Group, School of Biosciences, University of Kent, Canterbury, UK; h Infectious Disease Preparedness and One Health, Statens Serum Institut, Copenhagen, Denmark; i Istituto Zooprofilattico Sperimentale dell’Abruzzo e del Molise (IZS-Teramo), Teramo, Italy; j Department of Agricultural Sciences, University of Naples ‘Federico II’, Portici, Italy

**Keywords:** Bluetongue, domestic ruminants, seroprevalence, risk factors, meta-analysis, Africa

## Abstract

Bluetongue (BT) is a vector-borne disease affecting wild and domestic ruminants in many parts of the world. Although bluetongue virus (BTV) is widespread in ungulates in Africa, available epidemiological information on BT in this continent is limited. This systematic review and meta-analysis aimed to estimate the seroprevalence of BTV and summarize information on associated risk factors in domestic ruminants and camels in Africa. Systematic searches were conducted from the inception of the database to November 2022 on PubMed/MEDLINE, ScienceDirect, Web of Science, and Google/Google Scholar. Forty-four eligible publications were identified, published in the range from 1973 to 2020, and statistically analyzed. The pooled overall seroprevalence of BTV was 45.02% (95% confidence interval [CI]: 36.00-54.00%). The pooled seroprevalence was 49.70% (95% CI: 34.50-65.00%) in cattle, 47.00% (95% CI: 29.90-64.50%) in goats, 40.80% (95% CI: 19.60-63.90%) in camels, and 36.30% (95% CI: 29.00-44.90%) in sheep. The pooled seroprevalence decreased after 1990 and increased again after 2010. The highest pooled overall seroprevalence was found in the southeastern region, and the highest pooled overall seroprevalence was obtained by Competitive Enzyme-Linked Immunosorbent Assay. Finally, the seroprevalence in females (53.30%, 95% CI: 34.80-71.00%) was significantly higher than in males (28.10%, 95% CI: 17.40-40.30%) (*p* < 0.05). We showed that antibodies against BTV were common in African ruminants and camels. Monitoring the seroprevalence of BTV, as well as systematic and continuous surveillance of the *Culicoides* population, are encouraged to prevent and control the spread of BT.

## Introduction

Bluetongue (BT) is an infectious disease of ruminants and camels, which is transmitted by biting midges of the genus *Culicoides* (Verwoerd and Erasmus 2004). The etiologic agent is bluetongue virus (BTV), belonging to the genus *Orbivirus* of the family *Sedoreoviridae* (Mellor et al. 2009). It was first described in South Africa in 1876 when intensive European livestock production was introduced into the region (Henning 1956; Verwoerd 2009). To date, 36 serotypes for BTV have been described. These include 24 classical BTV serotypes and 12 that are considered atypical (Ries et al. 2020; 2021). BTV can infect wild and domestic animals (van der Sluijs et al. 2011); it is not known to affect humans. There are marked differences in the severity of the disease in different species or breeds of ruminants and in infection of the same species with different strains of the virus (Verwoerd and Erasmus 2004), with the clinical disease often more severe in sheep and white-tailed deer (Howerth et al. 1988; Drolet et al. 2013). Infections in cattle and goats are usually sub-clinical, and cattle are often considered amplifier hosts in endemic areas (Coetzee et al. 2012). Some strains, such as BTV-8 European strain, cause clinical disease in cattle, especially in naïve European areas (Elbers et al. 2008). Interestingly, BTV serotypes continue to emerge, notably BTV-3, which has been recently reported in Europe (Lorusso et al. 2017; Cappai et al. 2019; Boender et al. 2024; Voigt et al. 2024). It is noteworthy that BT causes significant economic losses even in the absence of clinical signs (van der Sluijs et al. 2016). In severe forms, clinical signs may include fever, depression, excessive salivation, nasal discharge, facial edema, hyperemia, ulceration of the oral mucosa, coronitis, and, in chronic forms, torticollis. In sheep, head and neck edema, lesions in endothelium and disseminated intravascular coagulation can cause cyanosis of the tongue, resulting in a ‘blue’ appearance that is reflected in the name of the disease, however relatively rarely reported (Maclachlan et al. 2009).

The field diagnosis of BT is usually performed based on epizootiology, vector abundance, clinical signs, and pathological lesions (Coetzee et al. 2014). At the laboratory level, different assays, based on either antibody or virus/RNA detection have been developed (Rojas et al. 2019). Serological techniques for the detection of anti-BTV antibodies can be divided into two main categories depending on whether they aim for serogroup determination (detection of the highly conserved VP7 protein specific for each serogroup) or serotype identification (neutralization techniques or detection of VP2 protein). Competitive ELISA is a widely used technique for serogroup determination (Rojas et al. 2019), and serum neutralization (SN) for BTV serotype identification. BTV isolation (VI) methods include VI in embryonated chicken eggs (ECEs) and in cell lines, coupled with antigen identification using reverse-transcription polymerase chain reaction (RT-PCR), real-time RT-PCR, immunofluorescence, sandwich enzyme-linked immunosorbent assay (sELISA), dot immunoperoxidase assay (DIA), virus neutralization, and immunohistochemistry (Saminathan et al. 2020). Direct detection of BTV remains mainly limited to research purposes, as the techniques are relatively expensive and challenging to implement and maintain for routine BT diagnosis and surveillance.

Being vector-borne, the spread and prevalence of BT depend on the presence of midges which in turn is closely linked with the environment. Currently, of the 1400 *Culicoides* species, about 30 are known to transmit BTV (Ander et al. 2012; Maheshwari 2012; Archana et al. 2016). Ambient temperature, humidity, seasonal rainfall, wind speed, and marshy areas in late summer and fall can provide favorable conditions for the vectors and for BTV transmission (Maclachlan and Mayo 2013; Benelli et al. 2017). However, in recent years, atypical serotypes have been observed with increasing frequency. For some of them, the horizontal transmission has been demonstrated (Maclachlan et al. 2019).

Africa is a vast continent consisting of 54 countries, covering an area of 30.3 million km^2^, with an estimated human population of 1.4 billion (AUNDES: Affairs United Nations Department of Economic and Social 2023). According to Food and Agriculture Organization (FAO), there were nearly 438 million goats, 384 million sheep, 356 million cattle, and 31 million camels in Africa (FAO 2023). BTV is endemic in Africa with e.g. South Africa having reported 22 serotypes that were detected across several time periods (Coetzee et al. 2012). In northern parts of Africa, BTV-1, −4, −10, and −12 have been detected in Egypt, BTV-1, −2, −4 and −9 in Libya, Algeria, Tunisia, and Morocco, BTV-8 and −6 in Tunisia, and BTV-8 in Morocco (Cêtre-Sossah et al. 2011; Drif et al. 2018; Lorusso et al. 2018; Ahmed et al. 2019; Mahmoud et al. 2019). However, to our knowledge, there is currently no systematic summary of the overall seroprevalence of BT in domestic ruminants in Africa. Therefore, we conducted this systematic review and meta-analysis of BTV seroprevalence and associated risk factors.

## Methods

### Search method and selection strategy

In accordance with the Preferred Reporting Items for Systematic Reviews and Meta-analyses (PRISMA) guidelines (Page et al. 2021), a systematic screening of existing literature was performed in the PubMed, Web of Science, ScienceDirect, and Google Scholar databases. The databases were searched from inception until November 2022 to identify all articles published in English or French providing information on the prevalence of BT in domestic ruminants in Africa. We used the terms ‘bluetongue disease’, ‘domestic ruminants’, ‘small ruminants’, ‘prevalence’, ‘epidemiology’, and ‘Africa’ individually or in combination as search terms in the databases. An initial selection based on the title and abstracts of the articles was made independently by two individuals. Articles selected by at least one reviewer were retrieved, and duplicates were removed. A second selection based on the full text was made by two dependent reviewers, and discrepancies were resolved through discussion.

**Figure 1.**
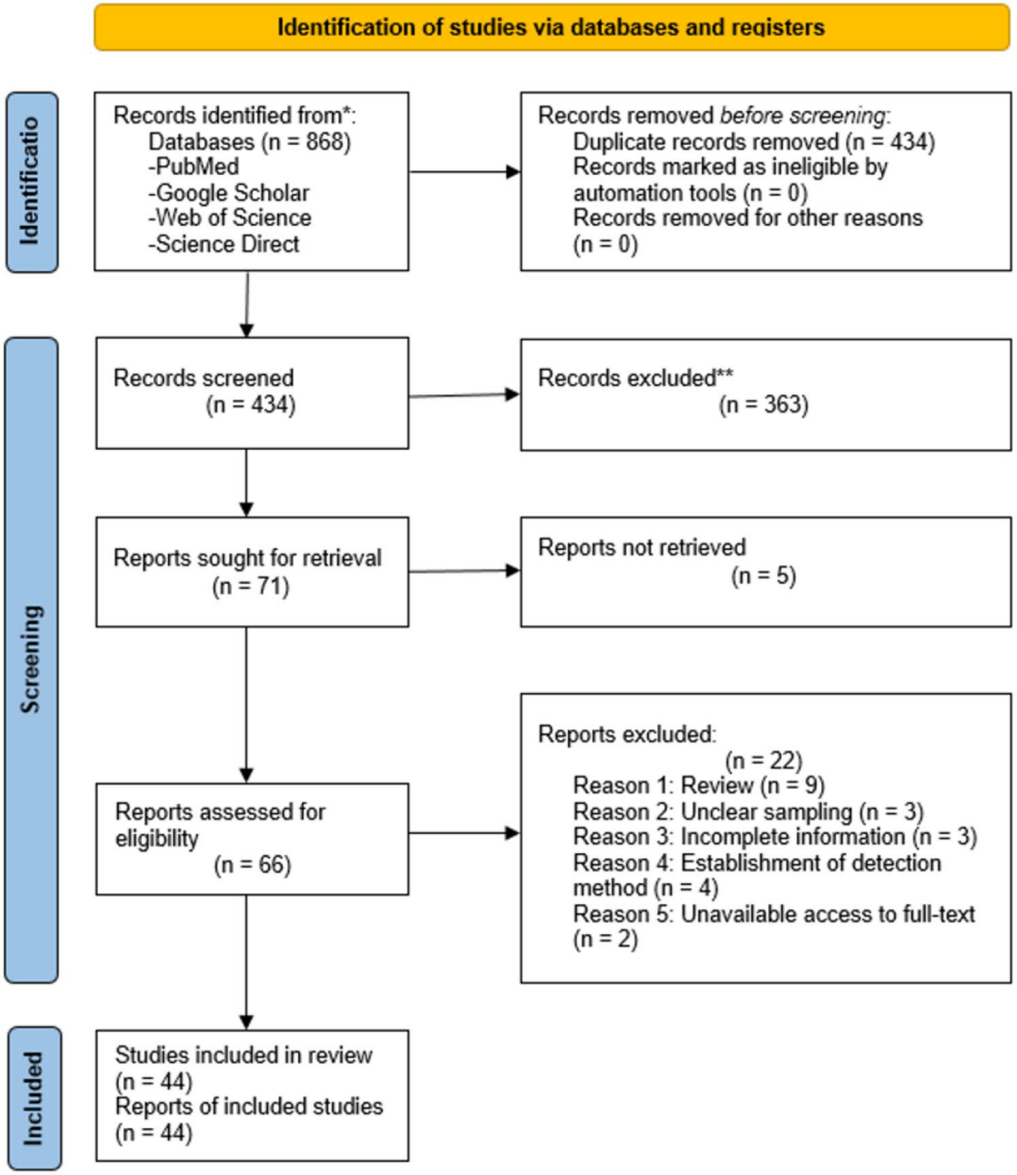
Flow chart for selecting studies.

The selection of eligible articles was based on previously established criteria: (1) articles dealing with the prevalence of BT or seroprevalence of BTV in domestic ruminants in Africa; (2) sample size greater than 30 animals (a decision made based on what can be considered a sufficient number from a statistical point of view); (3) number of positive animals for each species reported; (4) study design: cross-sectional study or surveillance report; (5) articles published in English or French. Articles that did not meet all five criteria were excluded. This generated a total of 44 articles that were included in the meta-analysis (Figure 1).

### Data extraction and quality assessment

Data were extracted by two independent, trained reviewers using a prewritten form, and discrepancies were resolved by a third reviewer. From each publication, we extracted the following information: first author’s name, year of publication, year of sampling, study area (based on the country in which the study was conducted), animal species, age, sex, diagnostic method, total number of tested animals, total number of species, total number of positive animals, and number of positive animals for each species. All included studies used serological methods. When multiple diagnostic tests were used, we considered only c-ELISA results to reduce heterogeneity.

The quality of selected studies was assessed using criteria based on the Grading of Recommendations Assessment, Development, and Evaluation (GRADE) (Atkins et al. 2004; Guyatt et al. 2008). GRADE is a framework for assessing quality of certainty of evidence and grading strength of recommendations. Each publication received one point for each satisfied criterion: (1) method clearly described; (2) sampling method clearly described; (3) indication of the sampling year; (4) random sample; (5) evaluation of at least four potential risk factors. Based on this score, we classified each publication as high quality (score = 4 or 5), medium quality (score = 2 or 3), or low quality (score = 0 or 1). The scores were not used for excluding publications; they are reported to guide future studies.

### Statistical analysis

We performed a meta-analysis of proportions (Miller 1978) using the ‘meta’ and ‘metafor’ packages available in the R software (version 4.2.2). Prior to the meta-analysis, we applied Freeman-Tukey double-arcsine transformation (PFT) to transform the proportions. This transformation is particularly well suited for normalizing and stabilizing the variance of the distribution (Freeman and Tukey 1950) (*dat<-escalc(measure="PFT", xi = xi, ni = ni, data = dat*). Because of the expected high heterogeneity in the meta-analysis of proportions, we chose a random effects model to assess the combined overall effect size and to perform subgroup analysis. Cochrane *Q* and *I^2^* statistics (expressed as *P* and *X^2^*, respectively) were used to assess and quantify heterogeneity. *I^2^* < 50% corresponded to low heterogeneity, whereas *I^2^* > 50% indicated high heterogeneity. Forest plots were used as summary graphics. Publication bias was assessed through Egger’s test and Funnel plots, whereas the stability of results was evaluated using stability analysis. Stability analysis consisted of assessing the impact of the deletion of a single article from the collection on the results obtained by considering the remaining papers.

We performed a subgroup analysis of the following potential risk factors: year of sampling (before 1990, 1990-2000, 2001-2010, and after 2010) (Gong et al. 2021; Liu et al. 2021), animal species (sheep, goat, cattle, and camel), age group (> 1 year and ≤ 1 year; this cut-off value of 1 year was chosen because the age categories in the different papers included in this study varied, and the cut-off of 1 year was suitable across the studies), sex (male and female), area (North, Northeast, South, Southeast, East, and West), detection methods (c-ELISA, Agar Gel Immunodiffusion (AGID), and others), and evidence quality (high, medium, and low; as defined in the previous section “Data extraction and quality assessment”). We also performed meta-regression to identify possible sources of heterogeneity; in this, we used year of sampling, area, detection method, sex, and animal species as co-variables. Further, we analyzed that data without outliers to evaluate if they affected the results.

## Results

Altogether 44 articles were included according to our inclusion criteria (Figure 1 and Table 1). Twenty-eight of them were identified as of high quality, whereas 13 and 3 were assessed as medium and low quality, respectively. All articles were kept in the study.

**Table 1.** Included studies of bluetongue virus in domestic animals in Africa.

First author	Year	Country	Detection method	species	Positive/Total samples	Quality
Cêtre-Sossah et al. (2011)	2011	Algeria	c-ELISA	Sheep, Goats, Cattle	460/755	High
Kardjadj et al. (2016)	2015	Algeria	c-ELISA	Sheep and Goats	20/150	Low
Kouri et al. (2018)	2018	Algeria	c-ELISA	Goats	70/105	Medium
Madani et al. (2011)	2011	Algeria	c-ELISA	Sheep, Goats, Cattle, Camel	333/1374	High
Hassine et al. (2017)	2017	Tunisia	c-ELISA	Camel	12/118	Medium
Davies and Walker (1974)	1974	Kenya	Other	Sheep, Goats, Cattle	537/1333	Medium
Zaher (2012)	2012	Egypt	c-ELISA	Sheep and Goats	98/200	Low
Gahn et al. (2022)	2022	Senegal	c-ELISA	Sheep and Goats	1023/1409	High
Simpson (1979)	1979	Botswana	AGID	Sheep, Goats, Cattle, Camel	941/1286	High
Simpson (1978)	1978	Botswana	AGID	Buffalo, Cattle	308/397	Medium
Ahmed et al. (2019)	2019	Egypt	c-ELISA	Cattle	94/227	High
Touil et al. (2012)	2012	Morocco	AGID	Camel	276/1392	High
Formenty et al. (1994)	1994	Ivory Coast	AGID	Sheep and Cattle	529/838	High
Selim et al. (2022)	2022	Egypt	c-ELISA	Camel	102/400	High
Abera et al. (2018)	2018	Ethiopia	c-ELISA	Sheep and Goats	129/422	High
Toye et al. (2013)	2013	Kenya	c-ELISA	Cattle	5/455	High
Drif et al. (2018)	2018	Morocco	c-ELISA	Camel	225/537	High
Ekue et al. (1985)	1985	Cameroon	AGID	Sheep, Goats and Cattle	85/126	High
Adam et al. (2014)	2014	Sudan	Other	Cattle	58/299	High
Davies (1978)	1978	Kenya	Other	Cattle	56/144	Medium
Chambaro et al. (2020)	2020	Zambia	Other	Cattle, Sheep and Goats	86/225	High
Eisa et al. (1979)	1979	Sudan	AGID	Sheep, Goats, Cattle, Camel	362/2142	High
Hafez and Ozawa (1981)	1981	Egypt	AGID	Sheep	10/31	Medium
Melaku et al. (2016)	2016	Ethiopia	AGID	Camel	92/120	Medium
Khaled et al. (2019)	2019	Egypt	c-ELISA	Sheep and Goats	112/607	Medium
Mulabbi et al. (2013)	2013	Uganda	c-ELISA	Goats	296/300	High
Mahmoud et al. (2019)	2019	Libya	c-ELISA	Sheep, Goats and Cattle	400/862	High
Andriamandimby et al. (2015)	2015	Madagascar	c-ELISA	Sheep, Goats and Cattle	4125/4393	High
Dione et al. (2022)	2022	Mali	c-ELISA	Sheep, Goats and Cattle	597/912	High
Lorusso et al. (2016)	2016	Mauritania	c-ELISA	Cattle and Camel	177/278	High
Dommergues et al. (2019)	2019	Mayotte	c-ELISA	Cattle	383/385	High
Daif et al. (2022)	2022	Morocco	c-ELISA	Sheep and Goats	689/1651	High
Ghirotti et al. (1991)	1991	Zambia	AGID	Cattle	5/214	High
Sailleau et al. (2012)	2012	Reunion Island	Other	Cattle	5/116	High
Weitzman et al. (1991)	1991	Niger	AGID	Sheep	16/70	Low
Lorusso et al. (2018)	2018	Tunisia	Other	Sheep, Goats and Cattle	31/62	High
Mahmoud and Khafagi (2014)	2014	Egypt	AGID	Sheep and Goats	219/1293	Medium
Elmahi et al. (2021)	2021	Sudan	c-ELISA	Camel	165/210	High
Abu Elzein (1986)	1986	Sudan	AGID	Cattle	147/261	Medium
Elfatih et al. (1987)	1987	Sudan	AGID	Cattle	67/161	Medium
Drif et al. (2014)	2014	Morocco	Other	Sheep and Cattle	128/436	High
Sana et al. (2022)	2022	Tunisia	c-ELISA	Sheep	1330/3314	Medium
Gordon et al. (2017)	2017	Zimbabwe	c-ELISA	Sheep and Cattle	115/209	High
Jørgensen et al. (1989)	1989	Zimbabwe	Other	Goats	512/724	Medium

c-ELISA, Competitive Enzyme-Linked Immunosorbent Assay; AGID, Agar Gel Immunodiffusion.

**Figure 2.**
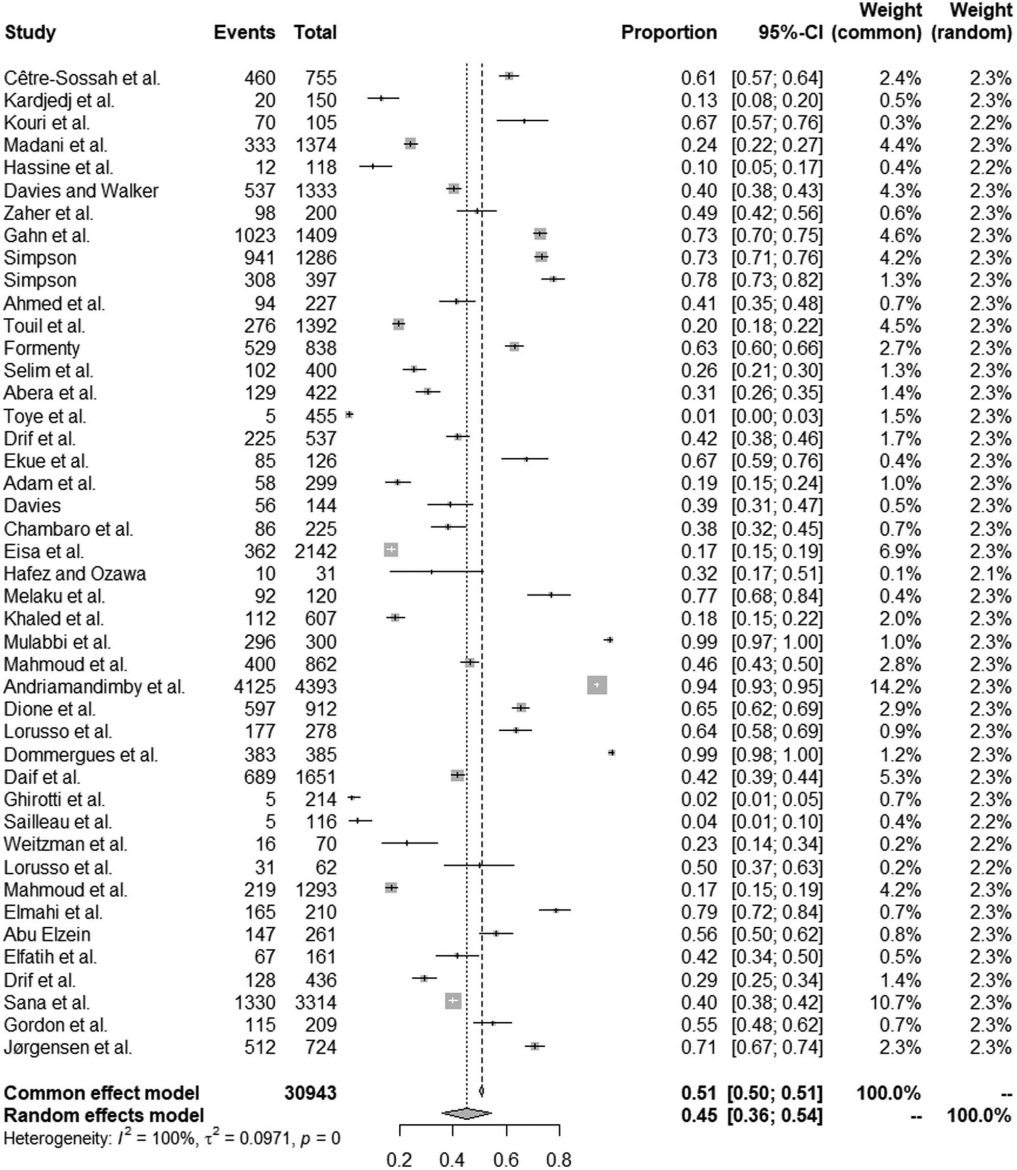
Forest plot of the seroprevalence of bluetongue virus in domestic animals among studies conducted in Africa.

**Figure 3.**
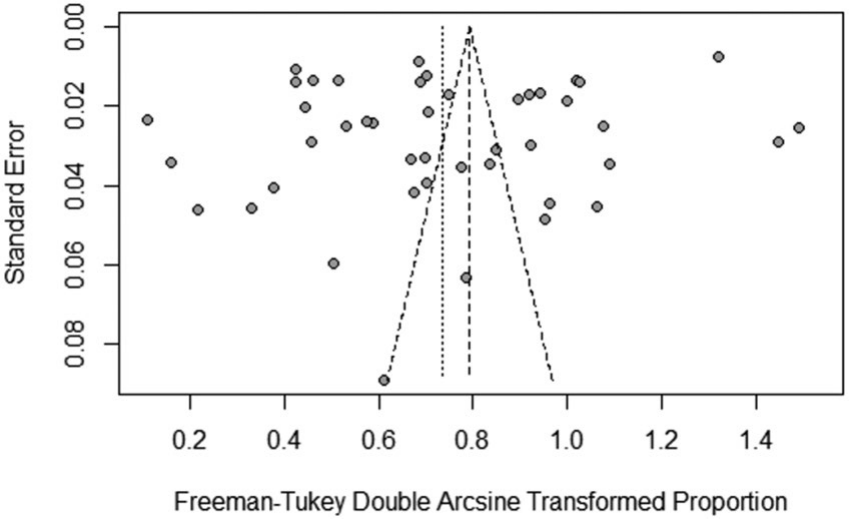
Funnel plot with 95% confidence limit intervals for the examination of publication bias.

The choice of using a random effects model was well justified by *P* and *I^2^* statistics which showed significantly high heterogeneity (*X^2^*=12819.0314 and *I^2^*=99.66%, *p* = 0.0001; Figure 2). Removal of the three poor quality studies did not affect the results, and we also did not identify publication bias in the selected studies according to visual inspection of the plot skewness (Figure 3), which was supported by Egger’s test (*t*= −1.22, *p* = 0.2284) (Table 2). We also considered funnel plots to assess publication bias in all subgroups (Figure S1). Since most of the surveyed studies did not clearly state whether they used random sampling, this may introduce sampling bias in our study as well. To address this issue, we performed a sensitivity analysis that indicated that the pooled seroprevalence results were not affected by removing single studies (Figure S2), which reinforces the robustness of our performed analysis.

**Table 2.** Egger’s test for publication bias.

Slope	bias	se.bias	T	df	p-value
0.0981	−6.3612	5.2044	−1.22	42	0.2284

**Figure 4.**
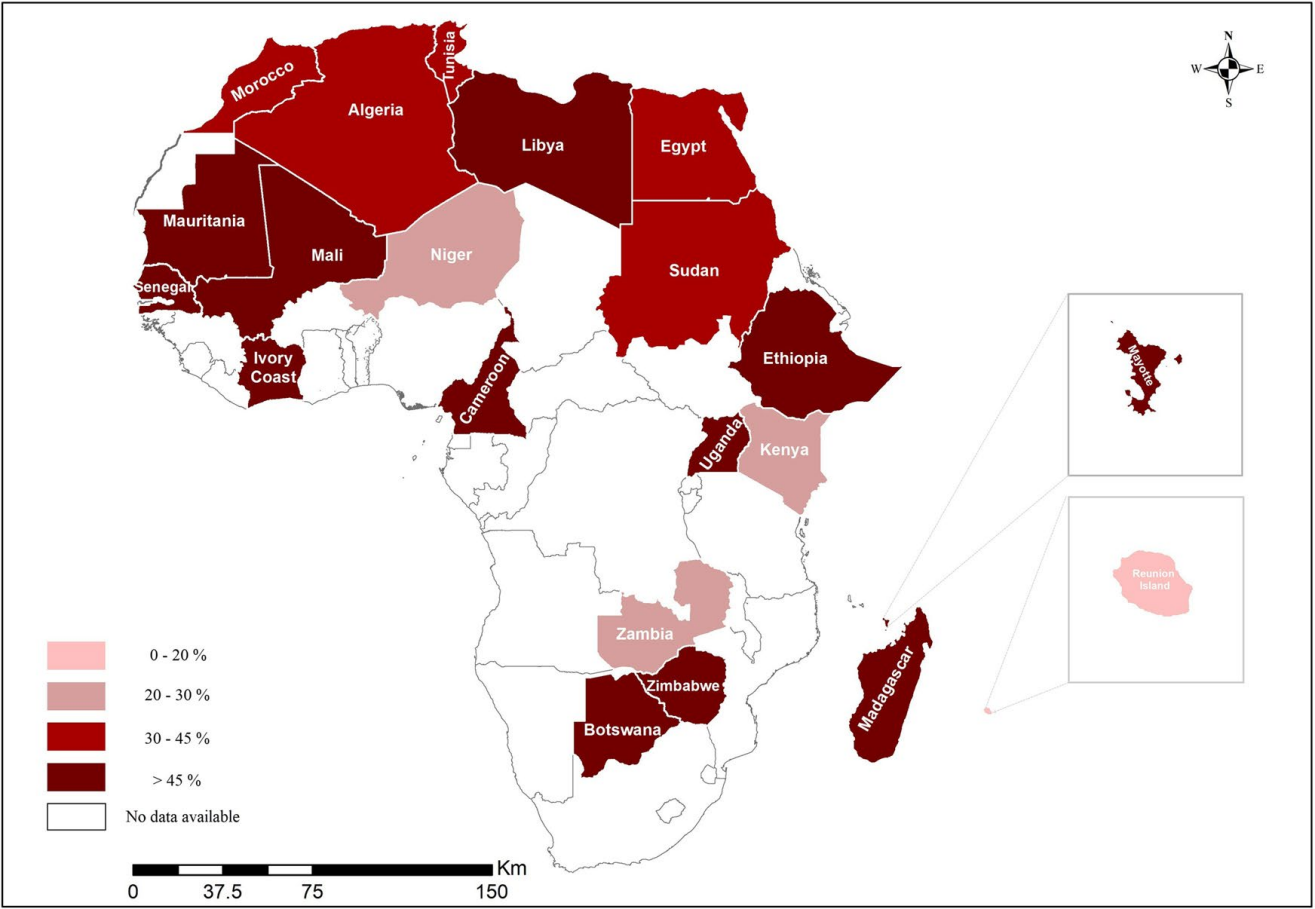
Bluetongue virus pooled seroprevalences estimates in domestic animals in countries in Africa.

The 44 analyzed publications included a total of 30,943 ruminants from 21 African countries. The overall pooled seroprevalence of BTV was 45.02% (95% confidence interval [CI]: 36.00-54.00%) (Figure 4). The results by potential risk factors for BTV seropositivity, including year of sampling, study area, animal species, sex, age, detection method, and study quality, are shown in Table 3. There was statistically significantly high heterogeneity in all subgroups, and pooled seroprevalence estimates for each subgroup were calculated using a random effects model.

**Table 3.** Pooled seroprevalence of bluetongue virus in domestic animals in Africa by potential risk factors.

Category	variable	No. of studies	No. of tested	No. of positive	% [95% CI]	Heterogeneity			Univariate meta regression		
						ꭕ^2^	*P*-value	*I^2^* (%)	*P*-value	*R^2^* (%)	*I^2^*-res (%)
**Sampling year**									0.8375	0.00	99.61
	1990 or before	7	6,057	2,726	50.20 [31.90-68.50]	1667.96	< .0001	99.6			
	1991-2000	7	1,879	964	41.90 [22.20-63.00]	443.14	< .0001	98.6			
	2001-2010	6	9,323 13,684	5,418	35.60 [9.80-67.20]	6386.30	< .0001	99.9			
	2011 or after	24		6,322	46.80 [34.00-59.90]	3657.31	< .0001	99.4			
**Area**									0.2752	1.18	99.70
	North	13	11,034	4151	38.10 [27.70-48.90]	784.41	<.0001	98.5			
	East	6	2,774	1,115	47.50 [14.80-81.50]	1387.86	<.0001	99.6			
	Northeast	11	5,831	1,434	35.10 [23.60-47.60]	630.97	<.0001	98.4			
	West	5	3,355	2,250	58.80 [41.20-75.30]	86.08	<.0001	95.4			
	South	6	3,055	1,967	50.90 [24.50-77.00]	673.22	<.0001	99.3			
	Southeast	3	4,894	4,513	71.80 [5.00-100]	607.73	<.0001	99.7			
**Species**									0.5776	0.00	99.43
	Sheep	20	10,947	4,330	36.30 [29.00-44.90]	1097.78	< .0001	98.3			
	Goats	16	3,377	1,999	47.00 [29.90-64.50]	1321.25	< .0001	98.9			
	Cattle	30	11,421	7,078	49.70 [34.50-65.00]	9531.50	< .0001	99.7			
	Small ruminant	8	6,256	3,349	46.50 [28.60-65.00]	1328.03	< .0001	99.5			
	Camel	9	2,625	805	40.80 [19.60-63.90]	627.19	< .0001	98.7			
**Sex**									0.0254*	18.96	98.58
	Female	9	3,783	1,977	53.30 [34.80-71.00]	470.13	< .0001	98.3			
	Male	7	1556	508	28.10 [17.40-40.30]	106.97	< .0001	94.4			
**Age**									0.0935	1.62	98.95
	< 1 year	21	7,601	2,393	30.70 [19.90-42.60]	1618.33	< .0001	98.8			
	> 1 year	16	7,545	3,841	46.20 [32.60-60.00]	1298.38	< .0001	98.8			
**Detection Methods**									0.3553	0.00	99.61
	AGID	13	8,331	3,057	42.10 [36.30-64.20]	2536.46	< .0001	99.5			
	C-ELISA	23	19,273	10,960	50.30 [36.30-64.20]	8329.60	< .0001	99.7			
	Other	8	3,339	1,413	34.90 [20.70-50.70]	469.20	< .0001	98.5			
**Study quality**									0.3158	0.00	99.62
	Low	3	420	134	27.40 [9.50-50.20]	56.94	< .0001	96.5			
	Middle	13	8,608	3,472	44.60 [31.50-58.00]	1208.95	< .0001	99.0			
	High	28	21,925	11,824	47.20 [34.50-60.10]	10869.79	< .0001	99.8			

c-ELISA, Competitive Enzyme-Linked Immunosorbent Assay; AGID, Agar Gel Immunodiffusion.

We show the differences based on geography in Figure 4. The highest seroprevalence in terms of area was 71.80% (95% CI: 5.00-100.00%, 4513/4894) in the Southeast, while the lowest one was 35.10% (95% CI: 23.60-47.60%, 1434/5831) in the Northeast; there was no statistically significant difference by areas (*p* = 0.2752). At the country level, the highest seroprevalence estimates were in Mayotte (99%, 95% CI: 98.00-100%, 383/385) and Uganda (99%, 95% CI: 97.00-100.00%, 296/300), and the lowest ones in Kenya (1%, 95% CI: 0.9-1.9%, 5/455), Zambia (2%, 95% CI: 1.82-2.18%, 5/214), and Reunion Island (4%, 95% CI: 0.5-4.5%, 5/116).

The seroprevalence of BTV was 50.20% (CI: 31.90-68.50%, 2726/6057) before 1990; it decreased to 41.90% (95% CI: 22.20-63.00%, 946/1879) in 1991-2000, and further to 35.60% (95% CI: 9.80-67.20%, 5414/9323) in 2000-2010, and then increased to 46.80% (95% CI: 34.00-59.50%, 6322/13684) after 2010. However, no statistically significant differences were found in terms of year of sampling (*p* = 0.8375) (Table 3).

The highest seroprevalence of 49.70% (95% CI: 34.50-65.00%, 7078/11421) was estimated in cattle, while the lowest of 36.30% (95% CI: 29.00-44.90%, 4330/10947) was found in sheep. Intermediate values of 47.00% (95% CI: 29.90-64.50%, 1999/3377) and 40.80% (95% CI: 19.60-63.90%, 805/2625) were estimated for goats and camels, respectively. These differences were not statistically significant (*p* > 0.05).

Pooled seroprevalence by age group suggested that older animals (> 1 year) had higher seroprevalence (46.20%, 95% CI: 32.60-60.00%, 3841/7545) than younger ones (30.70%, 95% CI: 19.90-42.60%, 2393/7601; Table 3), but this difference was not statistically significant (*p* > 0.05).

We detected a statistically significant difference (*p* < 0.05) in pooled seroprevalence by sex. Female animals had a pooled seroprevalence of 53.30% (95% CI: 34.80-71.00%, 1977/3783) compared to 28.10% (95% CI: 17.40-40.30%, 508/1556) in males (Table 3).

Finally, the pooled seroprevalence estimates did not differ significantly by the method used; pooled estimates were 50.30% (95% CI: 36.30-64.20%, 10960/19273) for ELISA, 42.10% (95% CI: 36.30-64.20%, 3057/8331) for AGID, and 34.90% (95% CI: 20.70-50.70%, 1413/3339) for the other methods used.

Subgroup analysis identified sex as the subgroup having the largest impact (*R^2^* =18.96% and a residual variation of 98.58%), while the other factors (i.e. sampling year, species, detection method, and study quality) did not have an impact (*R^2^*=0.0). Such findings were also confirmed by meta-regression analysis that showed that sex could be the main source of heterogeneity (*p* < 0.05).

## Discussion

BT is an animal disease with grave economic consequences, qualified by the World Organisation for Animal Health (WOAH) as a notifiable disease that should not be neglected. Worldwide economic losses due to BTV are approximately $3 billion/year (Rushton and Lyons 2015). Therefore, knowledge and understanding of the epidemiological situation of BTV are essential.

To our knowledge, this is the first systematic review and meta-analysis of BTV seroprevalence in domestic ruminants in Africa. The high overall seroprevalence in the animals examined is noteworthy in an area where livestock account for between 17% and 47% of the gross value of total agricultural production (Rakotoarisoa et al. 2011) and might represent a constraint to the development of this sector. The pooled seroprevalence reported in this study was higher than that recorded in China for small ruminants (19%) (Liu et al. 2021) and cattle (12.20%) (Gong et al. 2021). This could reflect differences in bioclimatic, monitoring and prevention methods. However, our results seemed to be close to the results reported in India (Hassani and Madadgar 2020; Rupner et al. 2020), who reported a prevalence of about 40% in the animals examined. The farming systems in India are likely to be relatively comparable to those on the African continent.

Several serological tests have been implemented for the detection of antibodies against BTV in African countries. WOAH recommends the use of c-ELISA in international trade, while AGID and SN are considered suitable in very limited circumstances (World Organisation for Animal Health 2021). In this work, the highest seroprevalence was found with c-ELISA, which is considered one of the most sensitive diagnostic tests for detecting antibodies to all BTV serotypes (Singh and Prasad 2009). Unfortunately, c-ELISA cannot distinguish between infection and vaccination and has the disadvantage of not identifying the BTV serotype. In contrast, AGID is relatively cheap and simple but has a relatively low sensitivity and specificity, as well as cross-reactions with other *Orbivirus* serogroups, especially epizootic hemorrhagic disease viruses, potentially leading to false positive results (Zhang et al. 2016). Molecular methods are currently the best approach for detecting viral RNA with high sensitivity and specificity. RT-PCR can detect circulating strains and determine the serotypes. However, the high cost of instruments and reagents associated with molecular methods limits their use, in comparison to the use of serological methods (Mayo et al. 2021).

In the subgroup analysis focusing on sampling year, BTV seroprevalence was higher in the 1990 and earlier. However, the estimated prevalence increased again after 2011. BTV is not only endemic but also indigenous to Africa and has been detected in South Africa, Ghana and Nigeria before the 1950s (Prasad et al. 2007). Prior to 1990, the local infectious disease surveillance and prevention systems were not very mature, and there were few studies conducted specifically to investigate the epidemiology of BT in different African countries. Moreover, AGID was mainly used during this time since it is a simple and inexpensive method, but one that cross-reacts with other *Orbiviruses* (Zhang et al. 2016). In addition, vaccination was neither developed nor widely available at that time, allowing transmission of BTV. As of 2010, the predominant diagnostic method has become c-ELISA, which is more sensitive and specific and detects antibodies as early as 6 days after infection (Kramps et al. 2008), which may explain the slight increase in reported seroprevalence estimates.

Our analysis suggests that among the investigated animals, species was not a relevant source of heterogeneity. However, cattle had the highest seroprevalence, which could be either due to the longevity of cattle compared with small ruminants or the presence of a competent vector and its host preference in their rearing areas (Portela Lobato et al. 2015). For example, it has been demonstrated that a high abundance of *Culicoides* vectors directly leads to a high BTV prevalence (Mellor et al. 2000; Tweedle and Mellor 2002; Scolamacchia et al. 2014; Kluiters et al. 2015; Malik et al. 2018). In addition, climatic and geographic conditions could be important factors responsible for the different seroprevalence estimates across the animal species as they influence the distribution of both livestock farms and the vectors. The virus replication in vectors depends on the environmental temperature, with temperatures below 12 °C being preventative (Mugabi et al. 2020). Cattle farming areas have a humid and temperate subtropical climate with high rainfall; therefore these areas are conducive to BTV survival and transmission. A subgroup analysis of geographic distribution revealed that BTV seroprevalence was positively correlated with climatic factors and *Culicoides* species distribution. The areas with highest seroprevalence estimates (Table 3) are characterized by humid subtropical, oceanic, equatorial, and savanna climates (Peel et al. 2007). These climatic biotopes can favor the survival of midges and the spread of BTV. Regarding all risk factor analyses, it is crucial to emphasize that the seroprevalence estimates have a wide CI which could be attributable to the small sample sizes of the studies carried out; future in-depth studies are necessary to clarify the epidemiological situation in this part of the continent.

Analysis of the effect of sex on BTV seroprevalence suggested that this factor could be a source of heterogeneity. In the present study, the BTV seroprevalence was statistically significantly higher in females. This might be explained by the fact that female mammals generally produce more robust immune responses and so may be more readily detected as seropositive. In addition, different ways of keeping animals of the two sexes, with females being kept in ways where they might be more likely to be bitten by midges. Currently, there are little data on the relationship between sex and infection by the BTV, but it seems that this is a relevant aspect that requires further investigation. Sex as a potential risk factor was quite rarely mentioned in the articles collected for this work, and thus there is higher uncertainty in these results. Importantly, it would be good to investigate both sex and age as risk factors, in combination with relevant animal husbandry factors. Our results showed that pooled seroprevalence appeared higher in the older age group, however, there was no statistically significant difference between the two age groups. However, the age groups were selected based on the other studies and suitability for the data, while e.g. the age reaching maturity for each species was not taken into consideration. Also, it would be interesting to investigate infections in very young animals to make inferences about vertical transmission.

The quality score given to articles was mainly affected by the lack of reporting on possible risk factors associated with BTV seropositivity. Moreover, the majority of publications did not include information on the presence of competent vectors in the study area. More studies are needed on BT in African countries, and these observations regarding quality can guide future studies and their reporting.

Comparable data are needed across countries and animal species, and harmonizing approaches should be encouraged. Importantly, possible risk factors for BTV seropositivity in domestic animals should be included in future epidemiological surveys to provide relevant information for effective monitoring and control plans. A One Health approach that includes surveillance of vectors as well as wildlife and possible reservoirs in the wild, while focusing on indirect effects on humans due to production losses in the food chains, could have a relevant impact to understand and control BTV.

We highlight four main aspects that could be biasing our meta-analysis: 1) the difficulty in accessing some data archives: many surveys carried out by governmental institutions are not available on digital databases; 2) some of the included studies did not provide all the information needed for subgroup analysis (e.g. rearing patterns and presence of BTV-competent *Culicoides* vectors); 3) the number of studies per country and area was very heterogeneous: for example, no data were available from Central Africa; 4) not all studies explicitly mentioned random sampling which could lead to sampling bias issues. Despite these limitations, the robust systematic approach used in this work yielded results that can be useful for planning future studies as well as monitoring and control of BT in the African countries and similar settings. We encourage active and passive veterinary and entomological surveillance programs using a One Health approach to be implemented, and more studies planned to identify relevant risk factors for BTV, in particular in cattle.

## Conclusion

We performed an extensive systematic review and meta-analysis of available literature and assessed the seroprevalence of BTV in African countries. BT is an epizootic in domestic animals in some countries and enzootic in other countries, with a relatively high prevalence, which could be a crucial impediment to the development of the livestock sector on the continent. The identified data gaps and knowledge gaps should be filled with surveillance and targeted studies. Various factors could affect the rate of BTV infection in different animal species, and more information on these is needed.

## Supplementary Material

Supplemental Material
