# Supplementary material for: Seroprevalence and risk factors of bluetongue virus in domestic cattle, sheep, goats and camels in Africa: a systematic review and meta-analysis
Source: Vet Q. 2024 Aug 30;44(1):1–12. doi: 10.1080/01652176.2024.2396118 (PMC11370698; doi:10.1080/01652176.2024.2396118)
Supplement: Supplemental Material [file TVEQ_A_2396118_SM8530.zip › Suppl_Fig/Figures Caption.docx]

**Supplementary Figure Captions**

**Supplementary Figure S1**: Funnel plots of subgroups. **a.** Funnel plot with 95% confidence intervals for the examination of publication bias of sampling year subgroup; **b.** Funnel plot with 95% confidence intervals for the examination of publication bias of area subgroups; **c**. Funnel plot with 95% confidence limit intervals for the examination of publication bias of species subgroup; **d.** Funnel plot with 95% confidence intervals for the examination of publication bias of sex subgroup; **e**. Funnel plot with 95% confidence intervals for the examination of publication bias of age subgroup; **f**. Funnel plot with 95% confidence intervals for the examination of publication bias of detection methods subgroup; **j.** Funnel plot with 95% confidence intervals for the examination of publication bias of quality subgroup.

**Supplementary Figure S2:** Sensitivity analysis. After removing one study at a time, the remaining studies were re-combined using a random-effects model to verify the impact of a single study on the overall results.
