# Supplementary figures and images for: Seroprevalence and risk factors of bluetongue virus in domestic cattle, sheep, goats and camels in Africa: a systematic review and meta-analysis
Source: Vet Q. 2024 Aug 30;44(1):1–12. doi: 10.1080/01652176.2024.2396118 (PMC11370698; doi:10.1080/01652176.2024.2396118)

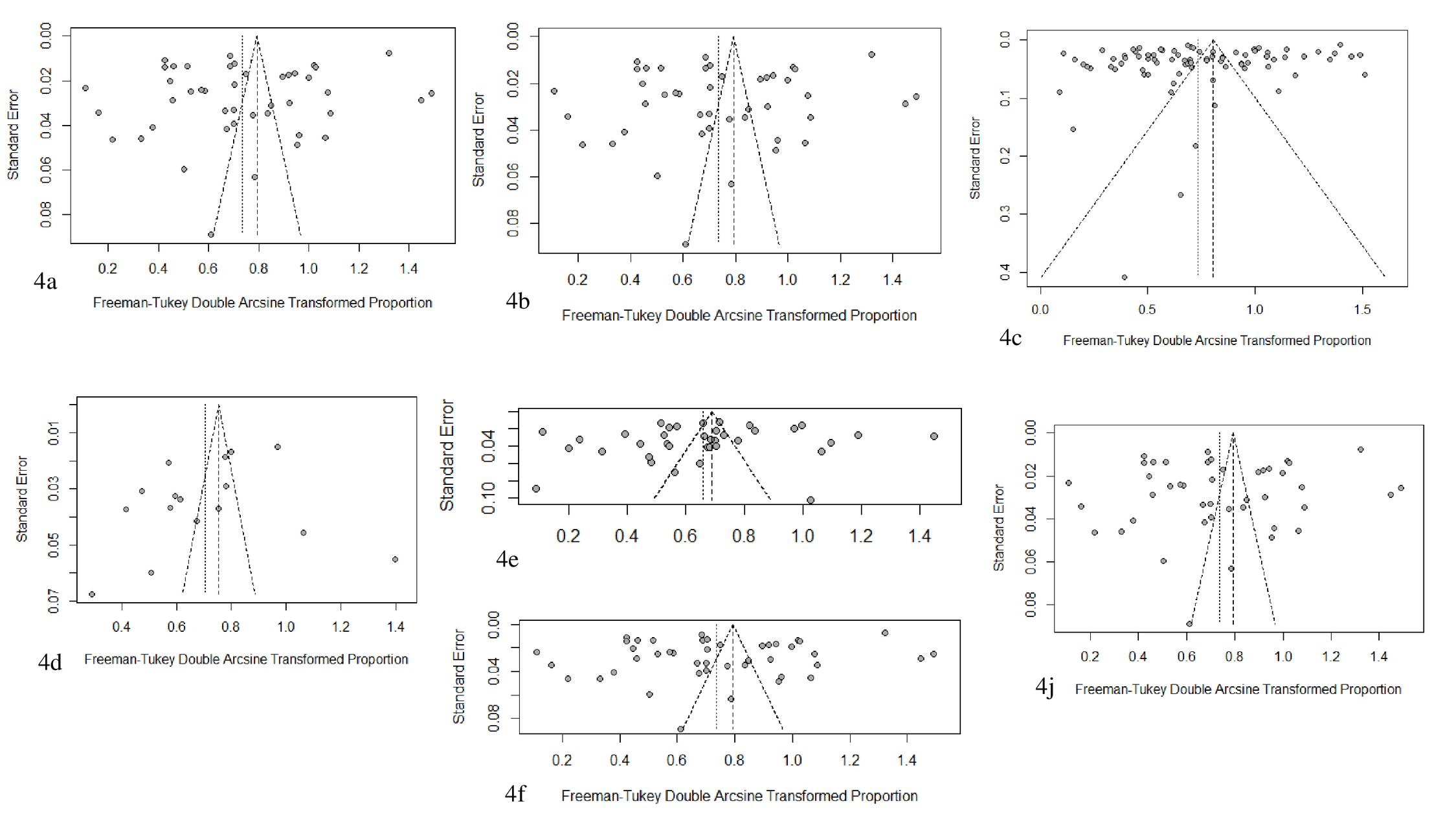

Supplement: Supplemental Material [file TVEQ_A_2396118_SM8530.zip › Suppl_Fig/Fig S1 Funnel plots by subgroup.tif]

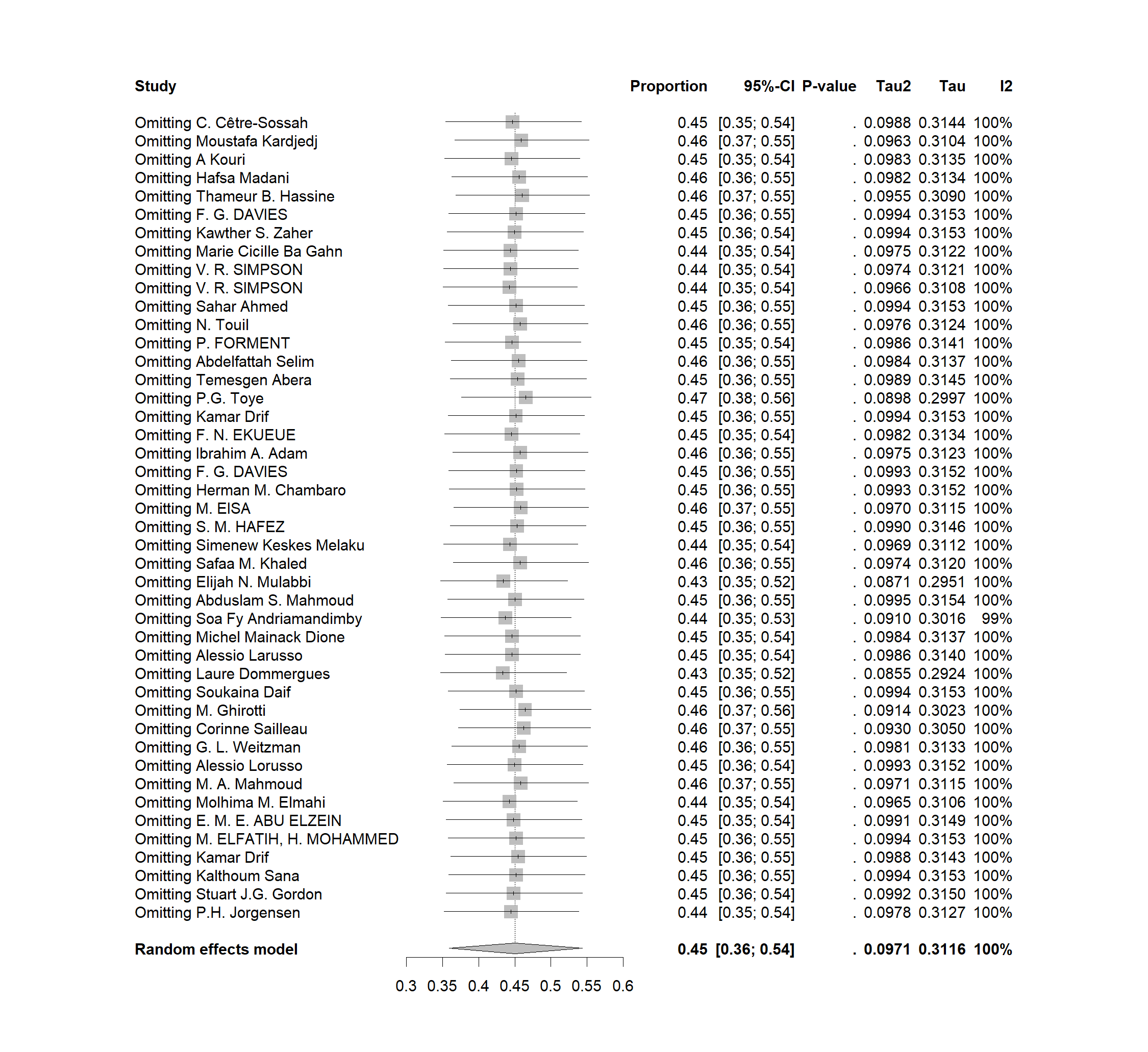

Supplement: Supplemental Material [file TVEQ_A_2396118_SM8530.zip › Suppl_Fig/Fig S2 Senstivity analysis.tiff]
